# “They will judge you more like a parent instead of like a health practitioner”: Perceptions and preferences of young people regarding sexual and reproductive health services in Southwest Nigeria

**DOI:** 10.1016/j.dialog.2022.100051

**Published:** 2022-09-29

**Authors:** Olujide Arije, Tintswalo Hlungwani, Jason Madan

**Affiliations:** a Institute of Public Health, Obafemi Awolowo University, Ile-Ife, Nigeria; b School of Public Health, University of Witwatersrand, Johannesburg, South Africa; c Warwick Medical School, University of Warwick, Warwick, UK

**Keywords:** Adolescents and young people, Young people's health, Sexual and reproductive health, Nigeria

## Abstract

Adolescents and young people (AYP) experience many barriers in the utilization of sexual and reproductive health (SRH) services. These barriers can be cultural, structural, personal or health worker-related. In this study, we explored the perceptions and preferences of AYP in receiving SRH services at public health facilities in a Nigerian setting. We conducted 16 focus group discussion (FGD) sessions with adolescents and young people allowing for maximum variation by sex (male, female), age (15–19 years and 20–24 years), and marital status (married and unmarried). We applied a thematic framework analysis to explore the data collected. Our findings included both positive and negative attitudes of health workers at public health facilities, non-involvement of AYP in activities relating to the planning, implementation, or evaluation of SRH programs for AYP, and non-awareness among AYP of some of the rights that AYP have with respect to SRH services in public health facilities. Many participants preferred younger health workers or those living within their neighborhood. Some older health workers were said to often act as (strict) parents, not health workers. We conclude that the role ascribed to ‘neighborhood’ nurses in this study is instructive and deserves more attention. Also, there is a need to increase the awareness of the young people about the type of SRH services they can obtain in the public health facilities, as well as, a need for health workers to be trained and retrained in providing SRH services to AYP.

## 1 Introduction

The perceptions of adolescents and young people (AYP) about sexual and reproductive health (SRH) services in health facilities are central to their utilization of these services. Freed *et al* [1] showed that adolescents’ perceptions about providers' behavior proved to be a strong predictor of visit satisfaction, while visit satisfaction was associated with intention to return. Though adolescents and young people are diverse, the most cross-cutting factors that make health services friendly to them are when service providers treat them with respect and assure that their confidentiality is protected [2]. Accessibility and acceptability are critical issues in providing high-quality sexual and reproductive health services to AYP [3].

However, gaps exist between the availability of, and adolescents’ need for general, reproductive, or counseling services [4]. Adolescents and young people experience many barriers in the utilization of services that should address their unique needs. These barriers can be cultural, structural, personal or health worker-related [5]. They include the sometimes judgmental and discriminatory attitude of health workers toward young people, especially with regard to issues about SRH [6]. Other barriers include the unpleasantness of facilities’ physical environment, unprofessional conduct and ill-informed professionals, inconvenient working hours, long waiting-time, expressing negative opinions about young people seeking reproductive health information, lack of privacy, and inadequate information [[7], [8], [9]]. Equally important are the financial inaccessibility to the services [10], and sometimes low self-efficacy of AYP to access SRH in public health facilities [[11], [12], [13]]. In sum, adolescents have difficulties using mainstream health services because of a perceived lack of respect, privacy, and confidentiality, fear of stigma and discrimination, and imposition of the moral values of healthcare providers [14]. This may be the explanation for why so few AYP have ever used the available sexual and reproductive health services [15].

**Fig. 1:**
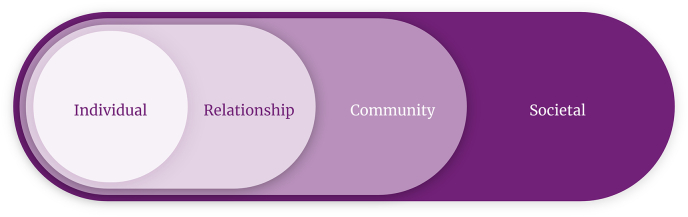
The Social Ecological Model. Source: adapted from the Centers for Disease Control and Prevention (CDC), The Social Ecological Model: A Framework for Prevention. https://www.cdc.gov/violenceprevention/publichealthissue/social-ecologicalmodel.html. (Retrieved 2 September 2022)

The barriers AYP face in accessing SRH services can be understood using the Social Ecological Model that acknowledges the inter-relational, multilayered, and hierarchical nature of the factors that drive health behaviours [16]. The model recognizes the layers of individual, relationship, community, and societal factors that influence human behaviour (Fig. 1). It gives a bird’s eye view of factors that drive AYP health behaviour and helps to conceptualize and contextualize possible solutions to problems identified.

Traditionally, primary health care delivery services in a country like Nigeria does not cater to the unique needs of AYP [10]. Instead, it has focused on providing comprehensive services that promote access to education about common health problems and what can be done to prevent and control them; maternal and child health care, including family planning; promotion of proper nutrition; immunization against major infectious diseases; an adequate supply of safe water; basic sanitation; prevention and control of locally endemic diseases; and appropriate treatment for common diseases and injuries [17,18]. There have been attempts to expand this scope to include services such as oral and mental health, and more recently, adolescent health services. It is important for health care planners to be aware that there are attributes that are somewhat unique to approaches for providing friendly services to AYP. In any case, hservices that are considered friendly to adolescents and youth are accessible, equitable, acceptable, appropriate, comprehensive, effective, and efficient.

In this regard, the concept of adolescent and youth-friendly health services (AYFHS) revolves around AYP’s peculiar needs that are often overlooked in many health systems and mainstream primary care services [14]. It is however not always feasible to build and operate dedicated services for adolescents. It is perhaps easier and more necessary to build on services that already exist [19]. Therefore, efforts need to be directed at making existing service delivery points adolescent-friendly. One way to do this is by getting AYP involved in the design, implementation, and evaluation of services targeting them [20].

As would be expected, personal preferences and perceptions about the quality of care play a critical role in the utilization of health services generally, and even more specifically for AYP. In recent years, efforts have increased to mainstream adolescents’ and young people’s health [21,22]. In the wake of the policy drive to promote adolescent health services in public health facilities in Nigeria, it is vital to study the perception and specific preferences of AYP concerning adolescent sexual and reproductive health services in public health facilities. Such an assessment will show the gaps and opportunities regarding accessibility, quality, affordability, and “user-friendliness” of adolescent health services at the primary care level. This research aims to provide an in-depth understanding of the perceptions and preferences of adolescents in receiving health care services at public health facilities. This study is a part of a larger study examining the demand and supply sides of sexual and reproductive services for adolescents in public health facilities in a Nigerian setting. This current study examines some aspects of the demand side and is also a precursor step in the design of a stated preferences study [23,24].

## 2 Methodology

### 2.1 Study design

This study used an exploratory qualitative method.

### 2.2 Description of the study area

The study location was Ogun State, which is one of the six states in the Southwest geopolitical zone of Nigeria. It is bordered by Lagos State and the Atlantic Ocean to the south, Oyo and Osun States to the north, Ondo State to the east, and the Republic of Benin to the west [25]. The predominant language of the people of Ogun State is Yoruba, with the individual sub-ethnic groups of the state speaking different dialects [25,26]. Adolescents and young people make up about 30% of the population of the State [27]. Ogun State has a total of 20 Local Government Areas (LGAs) [28]. This study was carried out in Abeokuta South LGA, a predominantly urban LGA, and Ijebu East, a predominantly rural LGA within the State. The use of rural and urban settings helps to explore differences among AYP in different settings.

### 2.3 Study population

The study population included self-identified married and unmarried female and male AYP in the two study LGAs. Inclusion criteria are female (15-24 years) and male (15-24 years), while the exclusion criterion was less than a year of residence within Southwest Nigeria.

### 2.4 Sampling technique

A series of focus group discussion (FGD) sessions were conducted in each of the study LGA to allow for a maximum variation of respondents in terms of sex (male, female), age (15-19 years and 20-24 years), and marital status (married and unmarried). Discussants were purposively selected to participate in the discussion sessions amongst persons who ordinarily live in study areas. Participants were identified and invited through community mobilizers to participate in the discussions. The groups' homogeneity was ensured in terms of age and sex for each session by having separate sessions for males and females of two age categories (15-19 years and 20-24 years), and marital status categories.

### 2.5 Study instrument

A semi-structured FGD guide was used to explore the perceptions of AYP about health services offered to AYP at public health facilities. The guide also included questions on their perceptions and preferences concerning accessibility and acceptability of sexual and reproductive health (SRH) services for young people in public health facilities. Barriers and facilitators of service utilization, privacy and confidentiality, equitable and rights-based services, and young people’s involvement were also included in the questions.

At the end of each session, participants were required to itemise and rank up to five priority areas for high-quality sexual and reproductive health services for AYP in public health facilities. This priority listing was done as part of tool development for a stated preferences study as earlier mentioned, and the findings are discussed elsewhere.

### 2.6 Data collection

Appropriately trained moderators and note-takers conducted each discussion session. Each of the sessions was audio-recorded after seeking permission from the participants. The maximum duration of any of the sessions was one hour. The FGDs were conducted in English and Yoruba languages since these were the predominant languages in the study area.

### 2.7 Data analysis

Recorded sessions were transcribed verbatim. Interviews conducted in a different language, specifically, Yoruba Language, were translated into the English Language during transcription. The audio recordings were transcribed by transcriptionists with multiple years of experience and who were familiar with English and Yoruba Languages. The transcripts were proofread by another set of transcriptionists different from those that did the original transcriptions as a form of quality check. We developed an analytical framework and codes (including a code dictionary) which were applied to all the transcripts from the discussion sessions to identify recurrent, dominant, and divergent opinions. The codebook was developed by AOO and revised by HTM. We applied thematic framework analysis to explore the interview transcripts within the analytic framework developed. We used the ATLAS.ti 9 software for analysis. We used codes to abstract meaning from discussants’ responses in a hierarchy of meaning in which tertiary codes represented overarching thematic areas of the study. Secondary codes represented sub-categories within these overarching thematic areas, and the primary codes identified the specific contents of transcripts that corresponded to each of the sub-categories [29]. The overarching thematic areas identified were SRH challenges of AYP, availability of SRH services, affordability of SRH services, equitable and rights-based services, competencies and motivation of health workers, acceptability of SRH services, privacy and confidentiality, and young people’s involvement. Coding was done by an external qualitative data analyst with up to five years of experience in coding qualitative research transcripts/data. The coding was revised by OA. Synthesis of and ‘memoing’ from the analyzed data was done by OA and the external analyst.

### 2.8 Ethical approval

This study was approved by the Human Research Ethics Committee of the University of the Witwatersrand (#M210315) and the Ogun State Primary Health Care Development Board (OGHECADEB) Ethics Committee (#OGPHC/021/008).

## 3 Result

### 3.1 Participant characteristics

A total of 16 FGDs were conducted in this study, eight in each study LGA disaggregated by age category, sex, and marital status (Table 1). The sessions had between 4 – 10 participants each. There was a nearly equal distribution of males and females, as well as, married and unmarried among all the participants. The majority of the respondents were Christians (72.7%) and of Yoruba ethnicity (90.4%). The participants from Abeokuta South LGA had a higher proportion of those with a tertiary level of education (75.9%) compared with Ijebu East LGA (21.4%).

**Table 1:** Background characteristics of participants

Background characteristics	Abeokuta-South	Ijebu-East	Total
	n=54 (%)	n=56 (%)	n=110 (%)
FGD Group			
15-19 years married female	4 (7.4)	5 (8.9)	9 (8.2)
15-19 years unmarried female	8 (14.8)	8 (14.3)	16 (14.5)
15-19 years married male	7 (13.0)	5 (8.9)	12 (10.9)
15-19 years unmarried female	10 (18.5)	8 (14.3)	18 (16.4)
20-24 years married female	4 (7.4)	6 (10.7)	10 (9.1)
20-24 years unmarried female	8 (14.8)	6 (10.7)	14 (12.7)
20-24 years married male	4 (7.4)	10 (17.9)	14 (12.7)
20-24 years unmarried female	9 (16.7)	8 (14.3)	17 (15.5)
Marital status			
Married	19 (35.2)	26 (46.4)	45 (40.9)
Single	35 (64.8)	30 (53.6)	65 (59.1)
Sex			
Male	30 (55.6)	31 (55.4)	61 (55.5)
Female	24 (44.4)	25 (44.6)	49 (44.5)
Religion			
Christianity	37 (68.5)	43 (76.8)	80 (72.7)
Islam	17 (31.5)	12 (21.4)	29 (26.4)
Traditional	0 (0.0)	1 (1.8)	1 (0.9)
Ethnicity			
Yoruba	50 (92.6)	50 (89.3)	100 (90.9)
Igbo	4 (7.4)	3 (5.4)	7 (6.4)
Others	0 (0.0)	3 (5.4)	3 (2.7)
Current level of education			
Primary	3 (5.6)	1 (1.8)	4 (3.6)
Secondary (junior)	2 (3.7)	5 (8.9)	7 (6.4)
Secondary (senior)	8 (14.8)	38 (67.9)	46 (41.8)
Tertiary	41 (75.9)	12 (21.4)	53 (48.2)

### 3.2 SRH challenges of AYP

Findings from this research showed a variety of perceived reproductive health challenges of AYP, including sexually transmitted infections (STI), HIV/AIDS, female genital cutting, irregular menstruation, and other menstruation-related problems, as well as unplanned pregnancy, and termination of pregnancy. Other reproductive health challenges according to the participants included *“pain in the private part”,* and *“womb damage”* (due to illegal abortion). Participants also talked about *“infection”.* Many of these participants opined that the infections could be “*toilet infection”* or itching in the private part.

The majority of the adolescents stated that adolescent reproductive health challenges are caused by sexual exposure that involves unprotected sex and multiple sexual partners, poor hygiene practices, as well as ignorance or lack of education about reproductive health issues. One of the participants said:

*Some of the youths are promiscuous, and some of them have multiple sexual partners and cannot do without having sexual intercourse in a day. All these are the causes of challenges for some of these youths and adolescents.* Rural married female 20–24 years

The social background of AYP that exposed them to child labor or that was associated with parental neglect was considered some of the underlying causes of SRH challenges in some AYP. Moreover, peer influences were said to lead to some of the SRH challenges in AYP. Some of the urban and older participants mentioned more specific problems like erectile dysfunction due to the use of drugs to enhance sexual performance. Also, there were some misconceptions that were revealed, such as gonorrhea being caused by urinating in the wrong spot or bathing inside the river.

### 3.3 Availability of SRH services

Concerning family planning services, many of the participants opined that they would use the services of formal hospitals or clinics, while a few others stated that they would prefer patent medicine vendors. Participants from both the rural and urban LGAs stated that young people use hospitals, clinics, or health centers if they thought they had a sexually transmitted infection. However, some young people preferred herbal concoctions from herb sellers, or drugs from patent medicine vendors and pharmacies. The reasons for preferring herbal medications included cheaper cost, less stress in accessing them, absence of waiting or protocols such as registration, and perhaps that they tend to cure faster. One participant felt also that herbalists would keep secrets safer than health workers who may discuss adolescents’ secrets with their colleagues or use the information as examples for others.

Furthermore, some participants felt some AYP would not go for any treatment, and just keep the matter to themselves*.* Some others were said to disclose to their parents so that they can help them find a solution. Other practices include self-medication and seeking information from peers. Only in one case did a participant mention AYP seeking information via the internet. Equally, another participant opined that some AYP would visit nurses, likely those living in their neighborhoods and not necessarily the health facility itself. The option of visiting a neighborhood nurse was also said to be cheaper than visiting the health facility. A participant said: “*… people prefer most to go to call some nurses around them to just take treatment, the reason why they prefer that too, most time, is based on the financial implication*” (Urban unmarried male in the 15 – 19-year age group). Some participants felt patent medicine vendors and pharmacies were considered more accessible and less demanding than health facilities because these places are situated near or within the community where young people live. In addition, the patent medicine vendor’s shop is preferred for STI services because the vendors attended to people on time. There were however participants who would prefer to go to the available health facilities because they felt the facilities offered better and more effective services. For instance, it was stated that there was an opportunity for carrying out laboratory tests at health facilities and that the health workers there were more knowledgeable than the other types of providers. A married male participant in the 15–19 years age group said: *“…they go there (*hospital*) because there are drugs that you will be given at the hospital that will cure the disease but the local herbalist might not know what caused the disease and he might even give treatment that might worsen the health condition”.*

### 3.4 Affordability of SRH services

Many of the participants revealed that family planning services are not offered for free at public health facilities. For example, an urban married male in the 20 – 24-year age group said “*There are some on family planning (*methods)*, if it is injection method, it may be given to them monthly and there is a certain amount that they pay for it, and there are some that are yearly there is a certain amount to pay too, but the amount they pay, I don't know.* However, some of the participants from the rural areas of the state opined that family planning and sexual reproductive health services and information are offered free of charge. One said: *“It* (family planning) *is done free of charge o, for example at the health centre, it was done freely for me* (rural married female in the 20 – 24-year age group).

Some participants who said the services were not free also stated the cost of the services ranged from ₦500 to ₦2000 (₦420≈$1, August 2021). A participant said, “*I have somebody that said she paid* ₦*1500”* (rural unmarried female in the 15 – 19-year age group), and “*Like a friend of mine used to say, some will say 500, some will say between 500 to 1000)* (urban married female in the 20 – 24-year age group). Some of the participants also stated that the cost of family planning and sexual reproductive health services was determined by the type of family planning method and the health facility that was being used. An urban married female in the 15 – 19-year age group said: *“…it depends on the hospital you go to, … like private hospitals they charge more”* Also, because of the easy availability of condoms in many neighborhood shops, participants found it easier to obtain condoms from there. A male participant said, *“Nowadays we do have people selling condoms around, so we can just go out and just get one and do what we want to do”.* Free condoms available in the public health facilities seems then to be a last resort as was indicated by a rural unmarried male in the 20 – 24-year age group who said: “*I will go there to collect* (condom) *maybe when I don’t have the money to buy it* (elsewhere)”*.*

### 3.5 Equitable and rights-based services

Some participants in this study were aware of some of the rights AYP have with respect to SRH services in public health facilities. An urban married older female participant indicated being informed by the health care providers of their right to freely walk into the health facility whenever they needed in order to get their SRH needs met. She said: “…*actually the place I’m talking about in my area, they actually tell us that we’re free to come in. If you go there and you ask them any question, they’re ready to attend to you…”.* Some also felt it was their right to obtain sexual and reproductive health services, as well as to receive prescribed drugs that are available at the facility at no cost. Nevertheless, several participants stated that many adolescents don’t access public health facilities for sexual and reproductive health services because they are not aware that they have such rights. An example is the comment of a rural married female in the 20 – 24-year age group who said: “*I am not aware of any right oh, and if there is, they should have contacted us and inform us of our right if there is anything they want to offer us*”. As such, some of the participants said they have not been using the services offered by public health facilities. In addition, some of the participants have experienced being told that they are too young to access SRH services. One of the participants said: *“…most of them* (health workers) *just push them (*AYP*) away, that they are too young. They pursue them away because they believe they’re still young to know about it (*sexuality matters/family planning*)”.* (Married female, 20–24 years age group).

### 3.6 Competencies and motivation of health workers

While many of the participants indicated that the services provided in public health facilities are welcoming, others reported both positive and negative attitudes of the health workers. Some of the positive attitudes of the health wealth workers included being educative, orderly, prompt, respectful, and friendly. In this regard, a participant said: “*… all these health workers in giving information, some of them actually appear like counselors, like guardians to some of the youth who are in that particular situation. So, some of them give or offer better solutions, developing solutions to the youth, not all of them reflect the angle that the youths have actually done the wrong thing…*” (urban unmarried male in the 20 – 24-year age group). Some of the negative comments about health care workers mentioned by participants included being aggressive, disrespectful, getting angry, unfriendly, harsh, judgmental, not prompt, and discrimination. One of the participants said: *“…when you go to them as a young man that you have so, so disease, they will judge you more like a parent instead of like a health practitioner, they will say why have you done this? Instead of them saying “oh you have done this,” you may need drugs, but they will be like ah! ah! why did you do this? You should not have done this…”* (urban unmarried male in the 20 – 24-year age group).

### 3.7 Acceptability of SRH services

Concerning the infrastructure in public health facilities, there were mixed perceptions about the cleanliness of the public health facilities. Many participants from both the rural and urban LGA felt the public health facilities where SRH services could be accessed were clean or neat. However, some participants perceived that the public facility environment was dirty; that is, having dirty beds and unclean bedsheets. A rural unmarried male in the 15 – 19-year age group said: “*the environment doesn’t really look like a hospital. It’s not okay, even the color is not fine”*. Concerning the acceptability of opening hours, participants generally felt the SRH services were open from Mondays through Fridays. Although some mentioned specific days like Wednesdays or Fridays which might be days when antenatal clinics are held in some health facilities. There were also varied perceptions about the opening hours of public health facilities. Some of the participants opined that the health facilities are open between the hours of seven AM and four PM. In contrast to this, few of the participants stated that public health facilities are open twenty-four hours.

### 3.8 Privacy and confidentiality

A number of participants recounted personal and other people’s experiences to buttress their sentiments that many health workers generally keep information provided to them confidential and as such find the health worker trustworthy. For instance, a rural unmarried female in the 15 – 19-year age group said: “*from the health workers that have I seen before when you discuss something with them, they don’t say it outside but also give advice on what to do”.* Another said: *for some, you can keep a secret with them and they will understand and keep it as confidential as possible* (urban married male in the 15 – 19-year age group). However, a participant felt health workers often don’t give enough weight to the confidential sexual and reproductive health issues raised by AYP. In response to such an attitude, some AYP are said to resort to being selective about the health workers they consult with, and what they discuss with them. An urban unmarried female in the 20 – 24-year age group said: “*maybe you will study the person first before you say you want to open up and it depends on how the person does that’s when you will know whether this person is worth it*”.

### 3.9 Young people’s involvement

Many of the participants reiterated that they are not involved in activities relating to the planning, implementation, or evaluation of SRH programs for AYP within the state. This is seen in the statement of an urban married female in the 15 – 19-year age group who said: “*I don’t think young people are even involved at all in the planning at the first stage because I have not come across any form of orientation or research work or question section that has to do with seeking our opinion in planning for such, then in the implementation they just put those things there and you just have to follow suit that is even if we want to follow suit…’.* One of the reasons offered for non-involvement of AYP is the belief that they are too young and therefore have nothing to contribute. The areas some of the participants reported that there has been the engagement of AYP in service provision are in health education, and sometimes community mobilization. A participant said: *“They do involve the youth if they have a program at the health center, they will call on someone to get some youths who could assist in whatever program they have* (rural married female in the 20 – 24-year age group).

## 4 Discussion

**Fig. 2:**
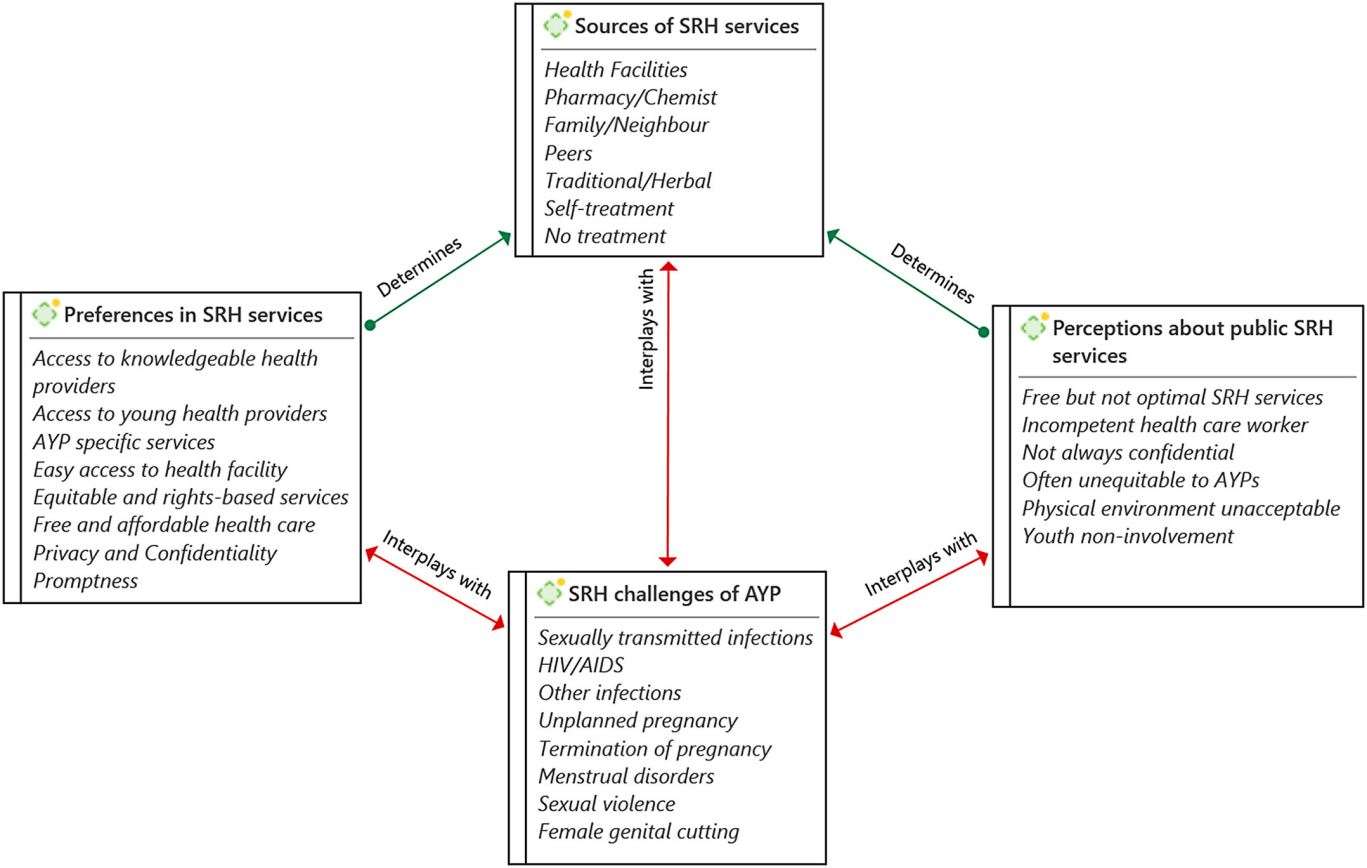
Interplay among SRH challenges of AYP, sources of SRH services, perceptions about public SRH services, and preferences in SRH services.

There has been an increase in the mainstreaming of sexual and reproductive health services for AYP in Nigeria [21,22]. Up-to-date research is required to understand and support national, regional, and local governments, and other stakeholders in the implementation of sexual and reproductive health policy and programs. In this study, we explored the perceptions and preferences of AYP about the current state of sexual and reproductive health in public health facilities in Ogun State, Southwest Nigeria. Fig. 2 shows that there is an interplay between where AYP choose to obtain SRH services, their perception about current services, their preferences for services, as well as the SRH challenges they are facing.

The most common SRH challenges that AYP faced in our study are similar to those experienced by AYP in similar, as well as other settings including STI, HIV/AIDS, unplanned pregnancy, termination of pregnancy, and poor knowledge about SRH issues [30]. The practice of alternative care for STIs seemed more common than seeking care in the health facility. The most preferred source of STI services seems to be traditionalists such as local herb sellers, herbalists, and traditional birth attendants. There are perhaps multiple reasons for this. It includes accessibility and affordability, as well as trust and relatability. These types of providers live within the same communities as the AYP, and more often than not charge less than formal care. Also, young people feel access to service at such places is less stressful compared to the hospital where protocols such as filling out forms or joining a queue need to be followed.

However, some of the factors driving the attitude of young people toward STI and its treatment are ignorance, and their young age, which makes them relatively inexperienced. This study confirms findings from other studies that indicate that health-seeking behavior and knowledge are different among the young and old [[31], [32], [33]]. The older AYP seemed more likely to take action about their SRH condition, while the younger were more likely to not open up, and as such not get the right help for their conditions. The younger ones likely only seek help at the health facilities when the issue is already complicated.

The role ascribed to ‘neighborhood’ nurses in this study is indeed instructive and deserves more attention. A neighborhood nurse is a health worker living in the same neighborhood as their potential clients. Many participants said they would be willing to trust nurses/health workers they know who live within their community. Also, visiting a neighborhood nurse was also said to be cheaper than visiting the health facility. The concept of neighborhood nursing is grounded in the promotion of a healthy community through collaborations with community members where they live [34]. This has been formalized in some settings, but it is still largely informal in the settings of this research.

Poor knowledge was one of the reasons given why young people prefer to access the patent medicine vendor shops rather than the hospitals for SRH services. Young people might assume some of the SRH problems as minor, and only visit the health facility when things have gotten worse. Also, AYP were said to find it easier to express themselves to patent medicine vendors and pharmacies than health workers at health facilities. Some studies have shown that fear is one of the barriers to the utilization of sexual and reproductive health services among AYP [35,36]. This includes the fear of embarrassment from the health worker, fear of meeting known faces in public health facilities, and fear of stigmatization. This is similar to the findings in this study. There is a need to train and retrain health workers in public health facilities in order to remove some of these barriers.

Despite the fact that condoms are available in some health facilities for free, many AYP would prefer to buy from outside vendors because of the ‘bureaucracy’ around obtaining condom. This is an area that needs improvement in the free condom program situated in health facilities. Policymakers and program designers also need to take cognizance that while acquiring a family planning method itself might be free, there might be other costs involved such as registration, consumables, and some indirect costs [37,38]. As seen in this study, opting to collect free condoms in a health facility may be a last resort for young people. Health workers in charge of the services need to demonstrate openness and friendliness to young people that come to collect condoms in order to remove any fear of being judged.

In this study, there seemed to be a perception that the older health workers were not modern or knowledgeable enough to know or understand the peculiarities of the modern AYP. While this is not necessarily correct, it underlies the need to make sure that health workers update themselves, or that avenues are created for health workers to have refresher training to keep abreast of the contemporary challenges of AYP [39]. However, the findings that some AYP prefer to confide in older health care providers compared to the younger ones is also enlightening. It shows that attitudinal change in one aspect or another is what may be required among older health workers.

## 5 Policy recommendations

The multilevel nature of the factors that drive AYP health behavior indicates that the solutions must also be proffered at these various levels. At the individual level, interventions that will positively influence AYP knowledge, beliefs, and attitudes are needed. This may include school-based intervention and community outreaches. We found in our study that many AYP do not have sufficient information about their rights to SRH services, or what to expect when they use the services. There is therefore a need to increase the awareness of the young people about the type of SRH services they can obtain in the public health facilities.

Next are the social networks and social support systems and relationships around AYP, including family support. Parents need to be targeted with health education that will change their attitudes and make them support the SRH of their children/wards. In a number of systematic reviews of interventions on parent-child communications about SRH, the interventions included were designed to enhance the frequency, depth, or quality of the content (including accurate information about STI and HIV) in parent–youth communication about sexual health [[40], [41], [42], [43]]. These interventions generally promoted early intervention at pre-adolescence, parental self-efficacy to talk about sex, parental knowledge of STIs risk and prevention, and addressed attitudes, beliefs, and norms that could be barriers to parental uptake of correct knowledge and practices about AYP sexuality. Some of the interventions increased parent-youth communications about engaging in risky sexual behaviours, and were associated with lower risky sexual behaviors among young people. Best practices for parent-based interventions include interventions that involve both the parents and their AYP, increase the self-efficacy of parents to talk to their AYP, and improve the knowledge of parents about sexual health or STI knowledge [42]. It is however important that these interventions should be started early enough before the AYP have become mature in their sexual decision-making [44]. Also, most of the African studies we found [[45], [46], [47], [48]] merely characterized parent-child communication about sexuality, implying a gap in the availability of interventions that demonstrate the effectiveness of parental communication in optimizing AYP SRH. This is an area for future research.

The community-level factors are represented by relationships between organizations, institutions, and resources, especially for health service provision. In this regard, health workers have a critical role in the provision of SRH services to AYP. An interesting area of future inquiry, and perhaps policy consideration, is the role of neighborhood nurses or health workers in promoting the SRH of AYP. Their availability within communities offers an opportunity for friendly and informed care for AYP. Other policy areas include training and retraining, especially for older health workers, in providing SRH services to AYP. This should include sensitization and attitudinal training that will help them become aware of their biases, and position them to provide services that are free from any moral judgment, both from cultural and perhaps religious point of veiw. There is as well a need for health workers to be trained about the rights of AYP to such services. Weiss et al [49] found that the lack of training in youth-friendly health services was associated with the feeling of incompetence to deliver such services among health workers. Soeters et al [50] proposed that AYP SRH interventions should have a human rights approach that includes values clarification and self-appraisal for health workers. They base their recommendations on the fact that health workers’ attitude toward AYP sexuality is often driven by their personal, cultural, and religious beliefs. Although this was in the context of AYP living with HIV, their proposal will find application in general AYP SRH.

Finally, the societal level represents organizational systems, as well as local, state, and national policies, strategies, and guidelines. According to our findings, young people are often not involved in the planning, implementation, and evaluation of SRH programs for AYP within the state. For the participants that felt that AYP were involved in the programs targeting them, it seems that inclusion is being interpreted as involvement. Obviously, the youths have to be included in the program that is targeted at them, but it should not be only as recipients or beneficiaries. Their involvement in carrying out the programs is still very low or non-existent and needs to be improved upon going into the future.

## 6 Conclusions

In this study, we provide some insights into how AYP perceive sexual and reproductive health services in public health facilities, as well as their preferences for care. In many cases, the reasons they have not used the available services is because of ignorance of these services, or perceptions of poor quality based on health workers’ attitudes, and cost. It is important to take cognizance that no matter what other approaches might be employed in reaching AYP, health facilities will remain central to these efforts [51]. Therefore, positive and innovative actions must continue in order to improve the quality of care that they offer AYP.

## Data availability

The data associated with this study can be provided upon reasonable request.

## Author contributions

OA, TH, and JM designed the study. OA conceived the study and provided training for the data collector and data analyst. OA, TH, and JM contributed to the design of the discussion guides. The code dictionary for data analysis, containing thematic codes and their definitions, was created by OA, and revised by TH. The coding of data done by a data analyst was revised by OA. All authors assisted with the interpretation of the results. OA wrote the article and all authors critically revised the manuscript.

## Funding

This research was supported by the Consortium for Advanced Research Training in Africa (CARTA). CARTA is jointly led by the African Population and Health Research Center and the University of the Witwatersrand and funded by the 10.13039/100000308Carnegie Corporation of New York (Grant No—G-19-57145), Sida (Grant No:54100113), Uppsala Monitoring Centre and the DELTAS Africa Initiative (Grant No: 107768/Z/15/Z). The DELTAS Africa Initiative is an independent funding scheme of the 10.13039/501100011858African Academy of Sciences (AAS)’s Alliance for Accelerating Excellence in Science in Africa (AESA) and supported by the New Partnership for Africa’s Development Planning and Coordinating Agency (NEPAD Agency) with funding from the 10.13039/100010269Wellcome Trust (UK) and the 10.13039/100013986UK government. The statements made and views expressed are solely the responsibility of the authors.

## Declaration of Competing Interest

The authors have no conflicts of interest relevant to this article to disclose.
